# Numerical Thermal Analysis and 2-D CFD Evaluation Model for An Ideal Cryogenic Regenerator

**DOI:** 10.3390/mi11040361

**Published:** 2020-03-30

**Authors:** Natheer Almtireen, Jürgen J. Brandner, Jan G. Korvink

**Affiliations:** Institute of Microstructure Technology (IMT), Karlsruhe Institute of Technology (KIT); 76344 Eggenstein-Leopoldshafen, Germany; Natheer.Almtireen@kit.edu (N.A.); Juergen.Brandner@kit.edu (J.J.B.)

**Keywords:** cryogenics, MATLAB^®^, numerical thermal analysis, cryocooler, regenerator, optimization, ANSYS Fluent

## Abstract

Regenerative cryocoolers such as Stirling, Gifford–McMahon, and pulse tube cryocoolers possess great merits such as small size, low cost, high reliability, and good cooling capacity. These merits led them to meet many IR and superconducting based application requirements. The regenerator is a vital element in these closed-cycle cryocoolers, but the overall performance depends strongly on the effectiveness of the regenerator. This paper presents a one-dimensional numerical analysis for the idealized thermal equations of the matrix and the working gas inside the regenerator. The algorithm predicts the temperature profiles for the gas during the heating and cooling periods, along with the matrix nodal temperatures. It examines the effect of the regenerator’s length and diameter, the matrix’s geometric parameters, the number of heat transfer units, and the volumetric flow rate, on the performance of an ideal regenerator. This paper proposes a 2D axisymmetric CFD model to evaluate the ideal regenerator model and to validate its findings.

## 1. Introduction

The growth in a large number of low-temperature applications led to huge developments in cryogenics and particularly cryocoolers over the past few decades. Top applications among these are IR imaging, i.e., micro-cryocoolers for IR imaging systems [1], superconductivity based systems, i.e., cooling of high temperature superconducting (HTS) motors [2], aerospace and military applications, cryosurgery, i.e., cryoablation of locations in the heart to treat heart arrhythmia [3], and many others. Some merits of these cryocoolers are comprised of durability, ruggedness, compactness, fast response, low vibration, and for the case of pulse tube cryocoolers (PTC), the absence of a mechanical displacer and relatively rapid cooling. Generally, cryocoolers utilize the oscillatory compression and expansion of a gas, commonly helium, within a closed volume to cool down an object. In general, any useful attempt to bring cryocoolers, especially pulse tube cryocoolers, down to the miniaturized scale requires a thorough study of each component’s working principle, since the cryocooler performance is highly dependent on the efficiency of each component involved in the thermodynamic cycle.

The regenerator is a vital component in any closed-cycle regenerative cryocooler, including the Stirling, Gifford–McMahon (G-M), and pulse tube machines. In pulse tube cryocoolers, the regenerator acts as a thermal storage component that transmits the pressure signal from the compressor to the pulse tube and other cryocooler components. It comprises a metallic porous medium that is subject to the periodic flow of the gas; where it is conventionally constructed of a thin hollow tube filled with mesh screens and/or metallic spheres, etc. The working principle involves thermal energy exchange between the working gas and the matrix material. To maintain considerable refrigeration, this matrix takes away the heat from the incoming gas during the heating phase and delivers it back for the returning gas during the cooling phase. The frequency of the gas flow can reach 100 Hz for a conventional PTC, but this should be significantly increased to serve any future miniaturized PTC design.

In 1816, Stirling introduced the regenerative heat exchanger in a hot air engine [4]. In 1927, Nusselt presented a mathematical analysis for a special case regenerator with infinite matrix heat capacity [4]. Iliffe used the ideal analyses of several German authors to perform a first-order graphical numerical analysis that accounted for time variations of the matrix temperature as early as 1948 [5]. One of the major advances happened in 1960, when Gifford and McMahon developed a small regenerative cryogenic refrigerator to cool infrared detectors and maser amplifiers, which moved cryogenics out of the laboratory to industry [4]. Kuriyama et al. [6] used rare-earth regenerative materials to achieve ultra-low temperatures with a G-M multi-stage cryocooler.

Analytical and numerical analyses of critical components are required for the development of more efficient pulse tube cryocoolers, to replace potentially conventional concepts in existing or emerging application fields [7,8,9,10]. In particular, these numerical analyses and thorough experimental works are very significant in providing better interpretation of the thermal interaction between the working gas and the matrix material in the regenerator. Radebaugh [11] introduced an optimization procedure for designing regenerators. The REGEN [12] series software developed at the National Institute of Standards and Technologies (NIST) was used by many researchers to study the regenerator performance for different matrix materials. The model in REGEN3.2 assumes sinusoidal mass flow at both ends of the regenerator, where the incoming gas temperature for both ends and the gas pressure are initially stated; also, the initial temperature profile is generally considered a linear profile. Further, the model sets the amplitude, frequency, and phase of the mass flow at both ends as input parameters. However, the equation that is used to describe the flow is not the conservation equation, but rather a predictive equation for pressure. Additionally, The gas velocity for all mesh cells is calculated simultaneously by solving a non-linear system; the remaining variables at each mesh cell are advanced in time explicitly. Hence, the model numerical approximation is considered semi-implicit [13]. On the other hand, REGEN3.3 is referred to as a fully-implicit model; since the numerical approximation involves discretization of the conservation of mass, momentum, and energy differential equations, whereas the non-linear system of equations for temperature, pressure, and mass flow at all mesh points are solved simultaneously by Newton iteration [13]. Other available software packages including SAGE^®^ and DELTAE^®^, which solve the conservation equations and hence describe the regenerator performance, are also often used. These numerical analyses have a range of complexity levels that depend on the assumptions employed and the degree of freedom used.

For all complexity levels, the design parameters of the regenerator, such as its diameter-to-length ratio, physical dimensions, pore structure, and matrix material parameters, impact the overall performance for any regenerative cryocooler. Normally, the design and/or optimization procedure of these parameters have been empirical, based on relatively rough lumped parameter considerations or the results of dimensional analysis. However, more complex numerical models should account for pressure drop, void volume, conduction losses, pressure variations, time-varying mass flow rates, time-varying inlet gas temperature, and temperature-dependent thermal properties. This paper presents a numerical approximation for the thermal interaction between the gas and the matrix material by only solving the energy conservation equations for both the matrix and the gas; the assumptions involve uniform one-dimensional longitudinal flow, with infinite radial thermal conduction and zero longitudinal thermal conduction in the matrix. It numerically solves the idealized general thermal equations for the working gas and the matrix based on finite-difference techniques and thus predicts the output temperature profiles for the working gas and the matrix material. Moreover, it studies the efficiency of the regenerator versus several design parameters of the regenerator. Finally, to validate the findings of the ideal regenerator model, the paper presents a 2D axisymmetric CFD model that solves the mass, momentum, and energy equations for the porous regenerator zone and provides more accurate results to compare it with the ideal regenerator model and examine its soundness.

## 2. Regenerator System

The regenerator is nearly analogous to a lossy spring that alternatively accepts energy and releases it when the excitation is removed. Another analogous model is a thermal flywheel accepting energy on the down-stroke and giving it back on the upstroke [14]. In the regenerator segment, the matrix is contained within a conventional steel tube housing in order to withstand the oscillatory high-pressure stream. The matrix should have an excellent thermal storage capacity with large heat transfer area, along with imposing a minimum pressure drop on the flow. Unfortunately, the last two characteristics are rather contradictory, since maximizing heat transfer area requires reducing the free flow area, which results in decreasing the gas volume and causing larger pressure drop. The advantages of regenerative heat exchangers can be listed as follows [14]:

- A large area for heat transfer can be obtained using inexpensive finely stacked material.
- The construction is relatively straightforward, and considerable savings can be made for the same heat exchanger duty.
- The regenerator tends to be self-cleaning due to the nature of the periodic flow reversals, and this is advantageous if contaminated gas is being processed.
- Well-designed regenerators can be achieved if proper design optimization procedures are in place.

However, the major disadvantage for static regenerative exchangers is the mixing of the hot and cold streams and is inescapable because of the carryover in flow switching [14]. This could disrupt the heat exchanging process and therefore deteriorate the cooling process; thus, great care should be taken when designing or constructing the regenerator element for optimum operation.

**Figure 1:**
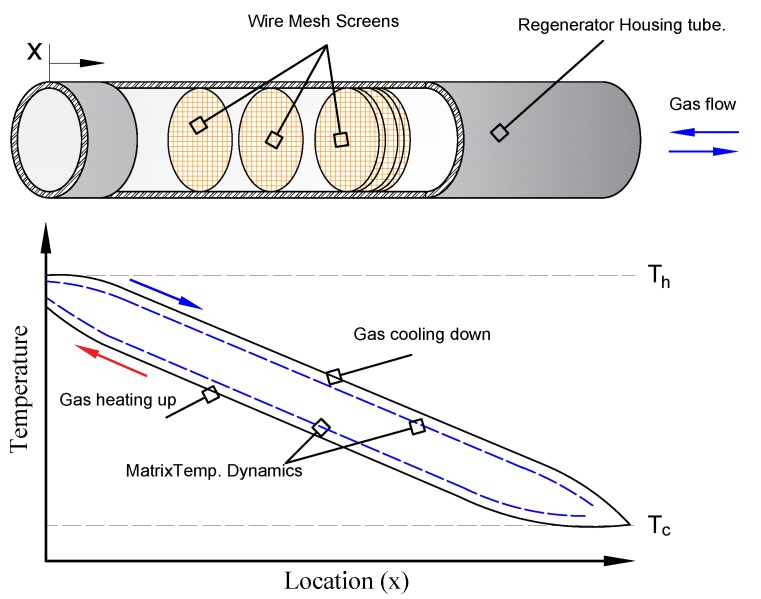
Expected matrix and gas temperature dynamics, where $T_{h}$ and $T_{c}$ are the respective hot and cold temperature levels.

In the regenerator, the temperatures of the gas and the pores vary with location and time. However, after several cycles of heating and cooling, a periodic steady state is achieved, as a result the nodal temperature at any location in the regenerator is equal to that temperature before/after one complete cycle at the same location. The expected temperature distributions for the gas and the matrix over one cycle are shown in Figure 1. It is worth mentioning that the cryocooler system itself is a closed system, in which no mass transfer out/to the system is allowed, and only energy transfer is permitted. Therefore, any indication of gas inflow/outflow refers to flow inside the system from one component to/from the regenerator.

### 2.1. Ideal Regenerator Model and Numerical Solution

The ideal regenerator can be interpreted as a thermodynamic object that receives the gas during the heating phase with low temperature T_c_ and releases it with high temperature T_h_. After the flow direction is reversed, the gas enters with temperature T_h_ and leaves with T_c_. In practice, the ideal regenerator case is impossible to achieve, where maintaining constant inlet/outlet temperature would need infinitely slow operation, or an infinite heat transfer coefficient, and/or a great heat transfer area. Furthermore, the heat capacity of the fluid and the matrix should be respectively zero and infinity. Moreover, the absence of a pressure drop would require a frictionless flow, while the absence of void volume would prevent the provision of flow passages through the matrix for the fluid to traverse [14].

The mathematical model used for this publication is based on simplified thermal energy conservation for the working gas and the matrix, as suggested by Ackermann [4]. The one-dimensional model is built on various assumptions: first, the mass flow and the working gas pressure through the regenerator are constant, and their magnitudes are equal during the heating and cooling periods, with no gas flow-mixing during these phases. In other words, the hot gas enters the regenerator at a constant temperature with constant and uniform velocity over the cross-sectional area; it stops when the flow is reversed, and the cold gas enters the regenerator with a constant temperature and flow rate. Second, the boundary temperature conditions for the working gas are constant for heating and cooling periods. Third, the fluid stored thermal energy is zero, with zero longitudinal matrix thermal conductivity. As a result, the matrix and fluid thermal equations are:

(1) $$\mathrm{Matrix}\mathrm{balance}\mathrm{equation}:h\delta A_{s}\left(T_{g}-T_{m}\right)={\left(\rho c_{p}\delta V\right)}_{m}\frac{\partial T_{m}}{\partial t},$$

(2) $$\mathrm{Fluid}\mathrm{balance}\mathrm{equation}:h\delta A_{s}\left(T_{g}-T_{m}\right)=-{\left(\rho c_{p}\delta V\right)}_{g}u_{x}\frac{\partial T_{g}}{\partial x}.$$

**Figure 2:**
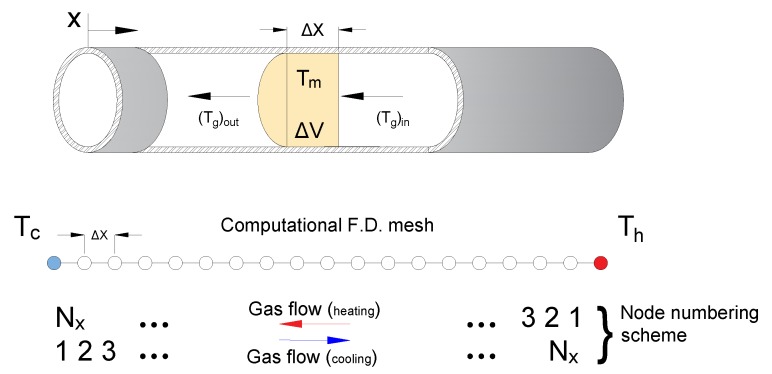
Numerical schematic for the regenerator during heating and cooling periods, where $N_{x}$ and $N_{t}$ are the total number of spatial and time elements and $T_{h}$ and $T_{c}$ are the hot and cold temperature levels.

Here, *h*, $A_{s}$, $T_{m}$, $T_{g}$, ρ, $u_{x}$, δV, and $c_{p}$ are, respectively, the convection heat transfer coefficient, matrix heat transfer area, matrix temperature, gas temperature, the density of the gas, the velocity of the gas, the control volume, and constant pressure specific heat capacity. The left-hand sides for both equations represent the convection heat transfer between the matrix material and the working gas, while the right-hand sides in Equations (1) and (2) represent the varying built-up heat in the matrix and the working gas. Equations (1) and (2) are then expressed in finite-element difference form as proposed by Ackermann [4], where the regenerator is divided into spatial elements and the heating and cooling periods are divided into sufficiently small time steps. Figure 2 shows the 1D computational mesh for the regenerator during heating and cooling periods.

The model is then converted to two linear algebraic equations, which are used to compute the nodal temperatures for the matrix and the gas, hence predicting the regenerator performance and finally yielding:

(3) $$\mathrm{Matrix}\mathrm{time}\mathrm{marching}\mathrm{scheme}:{\left(T_{g}\right)}_{i+1}^{j}={\left(T_{g}\right)}_{i}^{j}-K_{1}({\left(T_{g}\right)}_{i}^{j}+\left(T_{m}{)}_{i}^{j}\right),$$

(4) $$\mathrm{Fluid}\mathrm{time}\mathrm{marching}\mathrm{scheme}:{\left(T_{m}\right)}_{i}^{j+1}={\left(T_{g}\right)}_{i}^{j}+K_{2}({\left(T_{g}\right)}_{i}^{j}+\left(T_{m}{)}_{i}^{j}\right).$$

Here, *i* and *j* are the number of spatial and time nodes, and $K_{1}$ and $K_{2}$ are constants [4]:

$$\begin{array}{c} K_{1}=\left(\frac{{\left(\frac{hA_{s}}{{\left(\dot{m}c_{p}\right)}_{f}}\right)}_{\Delta x}}{1+{\left(\frac{hA_{s}}{{\left(Mc_{p}\right)}_{m}}\Delta t\right)}_{\Delta x}+\frac{1}{2}{\left(\frac{hA_{s}}{{\left(\dot{m}c_{p}\right)}_{f}}\right)}_{\Delta x}}\right)\\ K_{2}=\left(\frac{{\left(\frac{hA_{s}}{{\left(Mc_{p}\right)}_{m}}\Delta t\right)}_{\Delta x}}{1+{\left(\frac{hA_{s}}{{\left(Mc_{p}\right)}_{m}}\Delta t\right)}_{\Delta x}+\frac{1}{2}{\left(\frac{hA_{s}}{{\left(\dot{m}c_{p}\right)}_{f}}\right)}_{\Delta x}}\right) \end{array}$$

Here, $\dot{m}$, *M*, Δt, and Δx are the gas mass flow rate, the mass of the matrix material, and the time and the space differential elements, respectively. The equations are solved in a sequential fashion for each spatial and time node during both the heating and cooling periods. The numerical scheme assumes a linear distribution of temperature between $T_{h}$ and $T_{c}$ across the matrix. Furthermore, the fluid is assumed to enter the regenerator at $T_{h}$ for the heating period and $T_{c}$ for the cooling period. The MATLAB^®^ code calculates the nodal temperatures for both the matrix and gas during the heating period. The output spatial nodal temperatures for the matrix at the end of the heating period are then used as the input spatial nodal temperatures for the matrix at the start of the cooling period, and this can be expressed mathematically as:

(Tm)1:NxCooling1=(Tm)Nx:1HeatingNt

For the algorithm to converge to a solution, an increased number of time steps is needed; generally, the number of spatial nodes should be larger than the total number of heat transfer units (NTU). The relation between the regenerator inefficiency (Ie) and the spatial and time steps is discussed in Section 2.2 and Section 3.1.

The regenerator’s effectiveness term measures the performance of the heat exchange process relative to ideal heat exchanging conditions. It is defined as the ratio of the actual heat exchanged between fluids and media to the ideal exchanged heat if no temperature difference exists between the two fluids at any position. The expression of the regenerator’s effectiveness in the case of balanced inlet and outlet flows is [4]:

(5) $$\epsilon =\frac{T_{h,\;in}-{\bar{T}}_{h,\;out}}{T_{h,\;in}-T_{c,\;in}}=\frac{{\bar{T}}_{c,\;out}-T_{c,\;in}}{T_{h,\;in}-T_{c,\;in}}$$

where $T_{h,\;in}$, $T_{c,\;in}$, and $T_{c,\;out}$ are the temperature of the inlet heating fluid, the average outlet cooling fluid, and the temperature of the inlet cooling fluid, respectively. Generally, the performance of a cryogenic regenerator is defined rather by the inefficiency (Ie), where [4]:

(6) $$\mathrm{Inefficiency}\left(Ie\right)=1-\epsilon =1-\frac{T_{h,\;in}-{\bar{T}}_{h,\;out}}{T_{h,\;in}-T_{c,\;in}}$$

### 2.2. Model Convergence

The model converges to a solution under two conditions. During the heating cycle, the final matrix temperature should not be higher than the outlet heating fluid temperature. During the cooling period, the final fluid temperature is not higher than the cooling outlet fluid temperature. This can be summarized mathematically as:

$${\left[{\left(T_{m}\right)}_{N_{x}}^{N_{t}}\right]}_{Heating}≯{\left[{\left(T_{f}\right)}_{N_{x}}^{N_{t}}\right]}_{Heating},$$

$${\left[{\left(T_{f}\right)}_{N_{x}}^{N_{t}}\right]}_{Cooling}≯{\left[{\left(T_{m}\right)}_{N_{x}}^{N_{t}}\right]}_{Cooling}.$$

**Figure 3:**
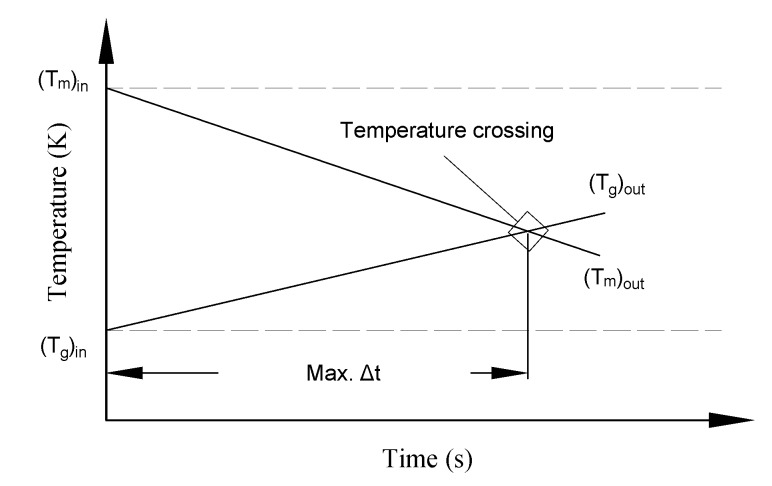
Convergence criteria for the numerical model during the cooling period.

From a mathematical point of view, the maximum time interval Δt, for each successive nodal calculation, is achieved when the output matrix and fluid temperatures are equal. If the outlet temperatures go beyond that, the crossing of the outlet temperatures causes a sign-reversal of the inlet temperature difference for the next calculation cycle and hence causes a divergence of the numerical solution. To prevent this crossover and thus divergence, the model must either set the time interval Δt to a minimal value or introduce a larger number of spatial nodes. Figure 3 illustrates the convergence criteria during the cooling period. The previous two conditions are applied to Equations (1) and (2) to deduce the reduced convergence formula: $K_{1}+K_{2}=1$.

**Figure 4:**
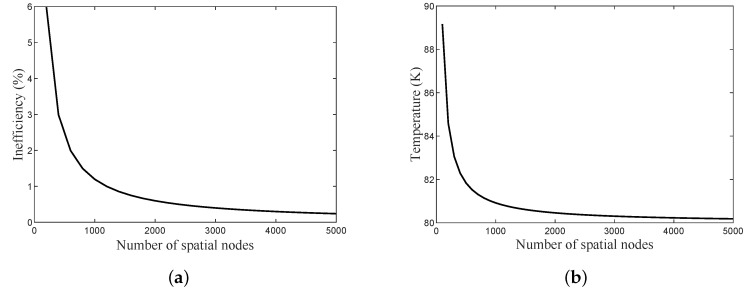
(**a**) Inefficiency (Ie) accuracy relative to the number of spatial nodes. (**b**) The accuracy in the final outlet gas temperature during the heating period both for the total number of heat transfer units (NTU_Total_) of 63 and $N_{t}$ of 200.

The number of spatial nodes is also significant in defining the accuracy of the computed variables. As the number of spatial nodes increases, the final outlet gas temperature during the heating period approaches the inlet gas temperature during the cooling period, thus decreasing inefficiency (Ie), as suggested by Equation (5). Figure 4a shows the effect of increasing the number of spatial nodes on the accuracy of the inefficiency as a calculated variable, and Figure 4b shows the effect of the number of spatial nodes on the final outlet gas temperature. It shows that the proposed finite difference numerical model requires at least 4000 nodes to achieve tolerable accuracy if $N_{t}$ is set to 200.

### 2.3. Model Application

The model is used to examine the effect of changing the regenerator dimensions (i.e., length and diameter) on the inefficiency (Ie). Second, it examines the implications of the matrix mesh screen parameters (i.e., wire diameter and mesh size) on the performance of the regenerator and its inefficiency. A particular case is highlighted, with the following parameters: a regenerator of 7.5 mm and 30 mm in diameter and length. The regenerator matrix mesh size is a 150×150 commercial phosphorous-bronze screen; the screen wire-diameter is 63 μm; and the screens have the same value as the regenerator diameter. Both the mesh size and wire diameter were used in the numerical model to calculate the hydraulic diameter for the screen, the porosity, the heat transfer coefficient, and the total heat transfer area $A_{s}$. Furthermore, if perfect stacking is assumed, the number of screens is set then to 238, and the cold and hot end temperatures are assumed to be 80 K and 300 K, respectively.

### 2.4. 2D Axisymmetric Regenerator Model

This section introduces a two-dimensional (2D) axisymmetric transient regenerator model to investigate the validity of the ideal regenerator model. The CFD model simulation was executed using ANSYS Fluent software since it is widely used for modeling and simulating fluid flows and heat transfer in various engineering problems. Although Fluent’s momentum, continuity, and energy governing equations are inaccessible for modification, Fluent allows users to program their user-defined functions (UDF) and to connect them to the primary model to modify boundary conditions, alter domain properties, or introduce variable sources or signals.

The proposed system consisted of three zones: hot temperature zone, cold temperature zone, and porous regenerator zone. The model assumed that the hot temperature zone was contracting at a constant rate, pushing the hot gas inside the regenerator, while the cold temperature zone expanded at the same rate to maintain constant overall volume during the cooling period. After that, the hot zone started to expand, and the cold zone contracted to retain their original volumes as the cold gas entered the regenerator zone from the other side when the flow reversed direction during the heating period. The hot and cold temperature zones were designed to ensure that gas entered at $T_{h}$ and $T_{c}$ during the cooling and hot periods, respectively.

**Figure 5:**
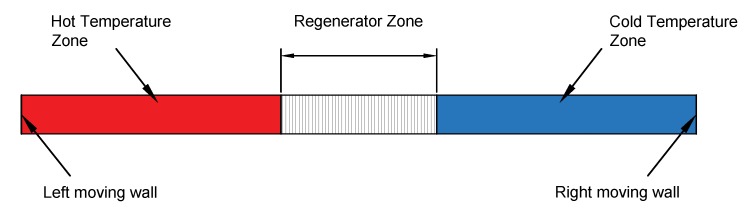
Two-dimensional regenerator model.

Figure 5 depicts the schematic diagram for the 2D model, where the dynamic mesh feature in Fluent, with C-language UDF, which defined the walls motion, were employed to enable the simultaneous motion of the left and right walls at constant speeds. The dynamic mesh feature allowed the user to create moving walls and deforming zones, which made any applications involving volume contraction and expansion modeled easily. In the dynamic mesh model, the layering option was employed to enable the addition or elimination of cells adjacent to a moving boundary based on the height of the layer adjacent to the moving surface in prismatic mesh zones [15]. The regenerator’s dimensions were the same as the highlighted case in Section 2.3. The porous medium was modeled by the addition of a momentum source term to the standard fluid flow equations. This source term created a pressure drop that was proportional to the fluid velocity, and it was composed of two parts: a viscous loss and an inertial loss term that is represented as [15]:

(7) $$\left(S_{i}\right)=\frac{\Delta p}{L}=-\left(\frac{\mu v_{i}}{\alpha }+C_{2}\frac{\rho \left|v\right|v_{i}}{2}\right)$$

where *L*, α, $C_{2}$, μ, and $v_{i}$ are the length of the regenerator, the permeability, the inertial resistance factor, the fluid viscosity, and the fluid velocity, respectively. The porous medium for this model was constructed of perfectly stacked #635 stainless steel screens with a wire diameter of 20.3 μm. The viscous and inertial resistance factors were applied to the porous zone, and their values were utilized from [16]. Landrum et al. estimated the hydrodynamic parameters of two fine-mesh porous materials (#325 and #635 mesh screens) by performing experiments, in which pressure variations across these mesh screens were measured under steady and oscillatory flows. Then, they simulated these experiments using a CFD model where the viscous and inertial resistances were iteratively adjusted until an agreement was reached between the experimental results and the simulated predictions [16].

The calculations reported here were performed using Intel^®^ Core™ i7-5500U cores @ 2.4 GHz with four cores. The settings for the model were axisymmetric and laminar, and the working fluid was helium as an ideal gas. The numerical discretization schemes were second-order for the pressure, second-order upwind for both the momentum and energy, and with implicit first-order transient time formulation. The simulated flow time was 80 s, with a 2 ms time step size and 10 iterations per time step. The space element size in the dynamic mesh zones was set carefully to avoid negative volume calculation errors.

## 3. Results and Discussion

### 3.1. Ideal Regenerator

**Figure 6:**
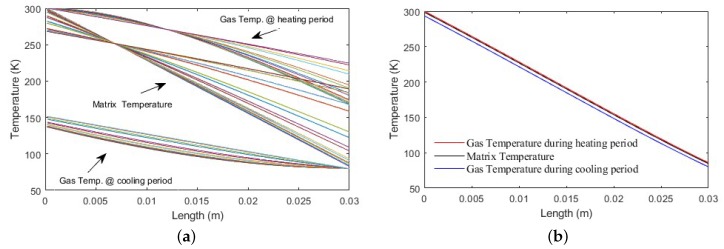
(**a**) Matrix and gas temperature variations during the heating and cooling period. (**b**) The final temperature profiles as they converge to a solution with increasing $N_{x}$ and $N_{t}$.

Figure 6a shows the gas and matrix temperature profiles during heating and cooling periods. It can be noticed that the temperature of the matrix material was increasing for all spatial elements during the heating period while the hot gas temperature dropped gradually as it crossed the regenerator, causing all the matrix elements to heat up. The cold gas was heated up as it passed the matrix, resulting in cooling the matrix. Figure 6b shows the algorithm after it converged and settled to a solution where the difference in temperature between the matrix and gas was minimal; this happened after increasing the number of time and/or space nodes, as described previously in Section 2.2.

**Figure 7:**
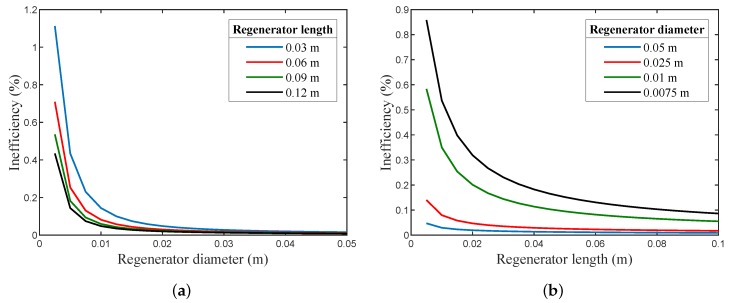
(**a**) The relation between the regenerator diameter and Ie. (**b**) The relation between the regenerator length and Ie (the matrix screen mesh size is 150×150 and has a wire diameter of 63 μm).

The dimensional and parametric analyses are of central importance for any efficient regenerator design. Figure 7a shows the relation between the regenerator diameter and its inefficiency (Ie), for several lengths, while Figure 7b illustrates the relation between the length of the regenerator and the inefficiency (Ie) for several regenerator diameters. These were based on the ideal case interpretation of the thermal interaction between the working gas and the matrix material; hence, it did not consider conduction or viscous losses. Furthermore, it assumed that the thermal properties for both the matrix and the working gas were constant with temperature variation inside the tube.

Although an abrupt change in the inefficiency was quite clear for diameters smaller than 0.01 m and lengths less than 0.03 m, the difference in the inefficiencies tended to be very small for diameter values above 0.025 m and lengths above 0.06 m. As a result, any attempt to miniaturize the regenerator element must take great care not to deteriorate its operation. The number of heat transfer units (NTU) is a non-dimensional parameter that expresses the regenerator’s heat transfer capacity. It is directly related to the size of the regenerator, its flow, and thermodynamic considerations. In a sense, if the NTU was large, the effectiveness of the regenerator would be high. It could be noticed that the inefficiency dropped with the increase in regenerator length and diameter, i.e., NTU increased as well, since the mass of the regenerator and its total heat transfer area increased as well. It is worth noticing that, in non-ideal regenerators, the axial conduction and viscous losses increases with larger lengths, and as a result, the inefficiency (Ie) also decrease.

Mesh screens can be found in a variety of sizes (i.e., 20×20 to 500×500). They are commonly used in many heat exchanging setups in research and industrial applications, which include air vents, cryogenics equipment, cryocoolers, coldheads, and heat pipes. The notation 20×20 refers to 20 wires/openings per inch.

**Figure 8:**
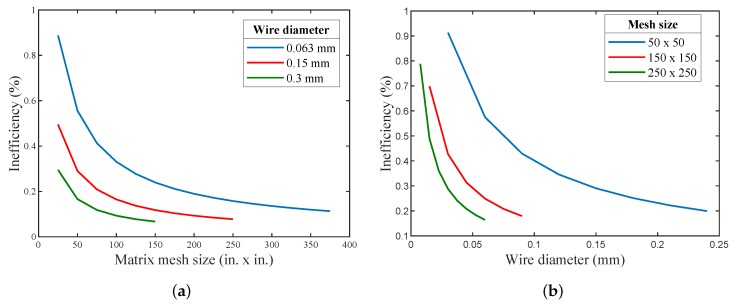
(**a**) The relation between the matrix mesh size and Ie. (**b**) The relation between the mesh wire diameter and Ie for several mesh sizes (for a regenerator of 7.5 mm diameter and 30 mm length).

Figure 8a exhibits the relation of Ie with matrix mesh size. It was evident that as the screen mesh size increased, i.e., the number of openings per inch increased, both the porosity and the hydraulic diameter decreased; and in contrast, the thermal penetration depth decreased, causing the total heat transfer area to increase, hence resulting in decreasing the regenerator’s inefficiency (Ie).

Figure 8b depicts the relation between the inefficiency (Ie) with the mesh screen wire diameter for several mesh sizes. It can be shown that the increase in the wire diameter decreased the inefficiency (Ie), since it decreased the porosity of the mesh screen, which was in turn inversely proportional to the mass of regenerator, hence leading to increasing the total heat transfer area. It is evident from Figure 8 that the regenerator’s inefficiency dropped with the increase in the wire diameter of the regenerator for the same mesh size, and this was also valid for different mesh screen sizes. Note that, as the wire diameter and mesh size increased, the pressure drop increased in the non-ideal case, since the gas free-flow area decreased.

**Figure 9:**
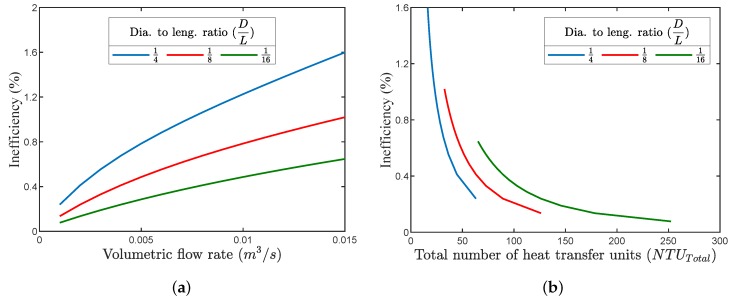
(**a**) Effect of increasing the volumetric flow rate on the regenerator’s inefficiency (Ie) for different regenerator diameter to length ratios. (**b**) Relation between NTU_Total_ and Ie for the same regenerator diameter to length ratios.

Figure 9a illustrates the relation between the volumetric flow rate on inefficiency for several regenerator diameter to length ratios (D/L). It can be noticed that, for an increased volumetric flow rate, the inefficiency increased due to a decrease in the total number of heat transfer units (NTU_Total_), as illustrated in Figure 9b. In order to maintain the inefficiency at the same level for increased flow rates, the matrix total heat transfer area had to be increased, which meant using a longer regenerator tube with a larger diameter. For the previously highlighted case with a volumetric flow rate of 0.001 $m^{3}/s$, NTU_Total_ of 63, $N_{x}=5000$, and $N_{t}=200$, the inefficiency (Ie) was found to be around 0.21% with 39.8 ≤ Re ≤ 365, where Re is the Reynolds’ number. This inefficiency level would cause a regenerator thermal loss of ∼ 0.39 W, which shows that adequate regenerator performance could still be achieved with such small dimensions.

### 3.2. 2D Regenerator Model

**Figure 10:**
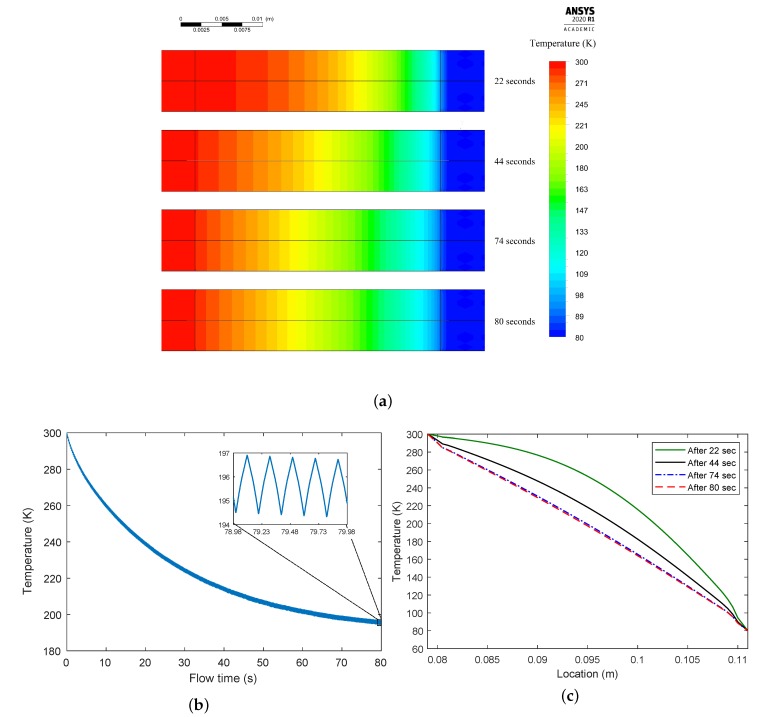
(**a**) Temperature contours after several flow times. (**b**) Average temperature versus flow time. (**c**) Temperature profiles across the regenerator’s center axis after different flow times.

The model assumed that the regenerator temperature was initially at $T_{h}$. Figure 10a shows CFD-predicted temperature distributions after four different flow times in the regenerator section. Figure 10b illustrates the regenerator average temperature profile versus flow time, and it shows the temperature dynamics during the heating and cooling periods as the cold and hot gas are flowing in and out of the regenerator. Moreover, it suggests a steady uniform temperature distribution after approximately 80 s in which an average temperature of 195 K was reached. The contour plot in Figure 10a proves this uniform distribution after a flow time of 80 s.

Figure 10c depicts the temperature profiles across the regenerator’s center axis after different flow times. It can be seen that the temperature became linearly distributed across the length of the regenerator. The temperature profile similarity between this and Figure 6b proved that the ideal regenerator model succeeded in describing the thermal interaction inside the regenerator.

**Figure 11:**
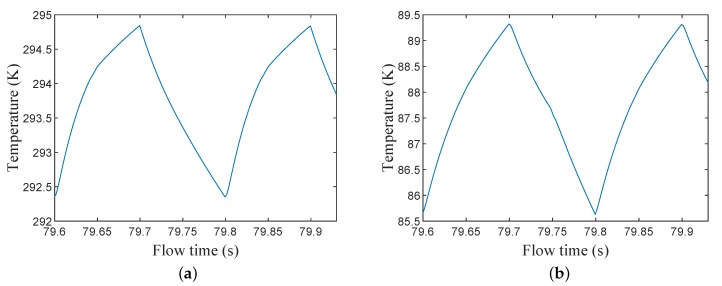
(**a**) Temperature changes during cooling and heating periods at the regenerator’s hot end. (**b**) Temperature changes during cooling and heating periods at the regenerator’s cold end.

Figure 11a,b show the temperature changes, during the heating and cooling periods, at the hot and cold ends, respectively. If a steady flow was assumed, the inefficiency could be calculated using Equations (5) and (6), and these were found to be 2.5% and 2.3% for the cooling and heating periods, respectively. The inefficiency value from the 2D axisymmetric model agreed with the findings in Figure 8b that decreasing the wire diameter would result in decreasing inefficiency. The 2D model proved that the stainless steel #635 mesh size was a good fit for a filler material for a miniature cryogenic regenerator.

Although the ideal regenerator model was a simplified model built on various assumptions and limitations, it not only worked in predicting the thermal behavior inside the regenerator, but also to roughly estimated the effect of the design parameter on the regenerator’s performance. However, for more consistent and accurate results, 2D or non-ideal models are required for any design or optimization attempts for the regenerator element. Future work will extend the model to cover the non-ideal case and then compare it to different regenerator CFD models.

## 4. Conclusions

A MATLAB^®^ code was developed to study the thermal interaction between the working gas and the matrix material in a regenerator element, a component of a closed-cycle regenerative cryocooler. The presented algorithm was a discretization of the ideal regenerator thermal equations. Furthermore, a 2D axisymmetric model was presented to examine the validity of the ideal model and evaluate its findings. This study illustrated the unsteady behavior of the gas and matrix inside the regenerator. It was observed that by increasing the number of spatial and time nodes, the convergence of the algorithm was enhanced. The paper also investigated the effect of regenerator length and diameter on its effectiveness and studied the relation between changing the matrix mesh screen size and its wire diameter on its inefficiency (Ie). It further investigated the implications of changing the volumetric flow rate and the number of heat transfer units on the performance of the regenerator. It was found that these parameters had a significant effect on its inefficiency (Ie) and that an optimum could be sought under given constraints. Finally, the temperature profiles from the 2D model were compared to the ideal model. Future work is to expand the model to consider other substantial losses in the non-ideal case.
